# Lower versus higher diagnostic criteria for the detection of gestational diabetes for reducing maternal and perinatal morbidity: study protocol for the GEMS randomised trial

**DOI:** 10.1186/s12884-020-03252-9

**Published:** 2020-09-18

**Authors:** Caroline A. Crowther, Lesley M. E. McCowan, Janet A. Rowan, Richard Edlin, Christopher J. D. McKinlay, for the GEMS Study Group

**Affiliations:** 1 Liggins Institute, The University of Auckland, Building 503, Level 2, 85 Park Road, Auckland Private Bag 92019, 1142 Auckland, New Zealand; 2 Department Obstetrics and Gynaecology, University of Auckland, Auckland, New Zealand; 3 Department of Obstetrics, National Women’s Health, Auckland City Hospital, Auckland, New Zealand; 4 School of Population Health, University of Auckland, Auckland, New Zealand

**Keywords:** Gestational diabetes mellitus, diagnostic threshold, large for gestational age, randomised trial

## Abstract

**Background:**

Gestational diabetes mellitus (GDM) has lifelong implications for the woman and her infant. Treatment reduces adverse maternal and perinatal outcomes although uncertainty remains about the optimal diagnostic criteria. The GEMS Trial aims to assess whether detection and treatment of women with GDM using the lower International Association of Diabetes in Pregnancy Study Groups diagnostic criteria compared with the higher criteria recommended in New Zealand reduces infant morbidity without increasing maternal morbidity.

**Methods:**

GEMS is a multicentre, randomised trial. Women with a singleton pregnancy at 24 to 34 weeks’ gestation are eligible who give written informed consent. Women are randomly allocated to the Lower Criteria Group or the Higher Criteria Group. Women with a normal OGTT by their allocated criteria receive routine care (Higher criteria: fasting plasma glucose < 5.5 mmol/L, AND 2 hour < 9.0 mmol/L; Lower criteria: fasting plasma glucose < 5.1 mmol/L, AND 1 hour < 10.0 mmol/L, AND 2 hour < 8.5 mmol/l). Women with GDM on OGTT by their allocated criteria receive standard care for GDM (Higher criteria: fasting plasma glucose ≥ 5.5 mmol/L, OR 2 hour ≥ 9.0 mmol/L; Lower criteria: fasting plasma glucose ≥ 5.1 mmol/L, OR 1 hour ≥ 10.0 mmol/L, OR 2 hour ≥ 8.5 mmol/L). The primary outcome is large for gestational age (birth weight > 90th centile). Secondary outcomes for the infant include a composite of serious outcomes, gestational age, anthropometry, Apgar score < 4 at 5 minutes, lung disease, use of respiratory support, hypoglycaemia, hyperbilirubinaemia, infection, and encephalopathy; and for the woman, a composite of serious outcomes, preeclampsia, induction of labour, mode of birth, weight gain, postpartum haemorrhage and infectious morbidity. A study with 4,158 women will detect an absolute difference of 2.9% in the proportion of large for gestational age infants from 10.0% using the lower criteria to 12.9% with the higher criteria.

**Discussion:**

The GEMS Trial will provide high-level evidence relevant for clinical practice. If use of the lower diagnostic criteria results in significantly fewer large for gestational age infants and/or improves maternal and perinatal outcomes these criteria should be recommended for diagnosis of gestational diabetes.

**Trial registration:**

Australian New Zealand Clinical Trials Registry registration number ACTRN12615000290594. Date registered: 27th March 2015.

## Background

Gestational diabetes mellitus (GDM) is a major and increasing health problem worldwide with rates varying depending on the population and diagnostic criteria used [1, 2]. Defined by the World Health Organisation as “carbohydrate intolerance resulting in hyperglycaemia with onset or first recognition during pregnancy” [3], GDM has immediate health risks for the women and her offspring [4] as well as lifelong implications for their health [5, 6], with adverse health effects continuing into the next generation [7–10].

### Treatment of GDM improves maternal and infant health

Convincing evidence from randomised trials shows that treating women with GDM with dietary and lifestyle advice, blood glucose monitoring and pharmacological therapy when needed, reduces the risk of serious perinatal outcomes, the chances of a large for gestational age infant, and improves maternal health-related quality of life [11–13]. These health gains are considered sufficient to justify the additional health service and personal costs of treatment for GDM [14].

### So what threshold for diagnosis and then treatment of GDM provides the greatest health benefits without harms?

Over the last 30 years there has been global controversy as to the precise degree of glucose intolerance required for the diagnosis of GDM, leading to the recommendations for use of several different diagnostic criteria worldwide [3, 15–20].

In New Zealand, the diagnostic criteria recommended for GDM, in use for over 30 years, are those developed through consensus by the Australasian Diabetes in Pregnancy Society (ADIPS) [15]. A diagnosis of GDM is made after a 75 g oral glucose tolerance test (OGTT) if the fasting plasma glucose is ≥ 5.5 mmol/L or 2-hour result is ≥ 9.0 mmol/L. The latest Ministry of Health clinical practice guidelines for GDM, “Screening, diagnosis and management of gestational diabetes in New Zealand,” endorse the continued use of these diagnostic criteria [21].

The Hyperglycaemia and Adverse Pregnancy Outcomes (HAPO) cohort study [22] confirmed a strong, continuous positive association between maternal glucose concentration and infant birthweight and cord-blood C-peptide levels, a marker for fetal hyperinsulinaemia. There was, however, no obvious threshold at which these risks increased [22]. Following extensive additional analyses of the HAPO Study, the International Association of Diabetes in Pregnancy Study Groups (IADPSG) recommended new, consensus based, diagnostic criteria for GDM [19] with a 75 g OGTT at 24 to 28 weeks’ gestation. Using these criteria, GDM is diagnosed if the fasting plasma glucose concentration is ≥ 5.1 mmol/L, or 1-hour result ≥ 10.0 mmol/L, or 2-hour result ≥ 8.5 mmol/L.

There has been considerable uncertainty [16, 18, 23] and significant ongoing debate [24–27] as to whether to adopt the IADPSG diagnostic criteria. A major concern is the greatly increased proportion of pregnant women who would be diagnosed with GDM. Although the exact increase in rate will vary by country, estimates for New Zealand [28] and similar populations suggest a two to three fold increase. Using the average HAPO rate of 17.8%, adopting the new criteria in New Zealand would more than double [28] the number of women diagnosed with GDM each year from 5,500 to over 11,000 women [2, 22]. For women ‘labelled’ as GDM there are personal costs and potential harms from increased obstetric and neonatal interventions, such as induction of labour, caesarean birth, and increased surveillance for and treatment of neonatal hypoglycaemia. Nevertheless, applying the IADPSG lower diagnostic threshold may reduce serious maternal and/or perinatal complications and later metabolic disease in both women and their offspring.

### Systematic review of diagnostic criteria for detection of GDM in women

Systematic review of the literature shows there have been no randomised trials to assess whether the IADPSG recommendations for detection of GDM [19], compared with the detection thresholds currently in use in New Zealand [15, 21] reduce the known risks for women with GDM and their infants, without increasing harms. With this lack of evidence from randomised trials to guide recommendations the Ministry of Health Clinical Practice Guidelines “Screening, diagnosis and management of gestational diabetes in New Zealand” identified the threshold for detecting GDM as an area of priority for further research [21].

### Aims and objectives of the GEMS Trial

The GEMS Trial will assess, based on OGTT results at 24 to 28 weeks’ gestation, whether detection and treatment of women with GDM using the lower IADPSG criteria [19] compared with the higher criteria currently recommended and used in New Zealand [15, 21], reduces significant perinatal morbidity without increased maternal risk, and to determine the health service utilisation.

### GEMS Trial Hypotheses

The primary hypothesis of the trial is that compared with the current recommended, higher New Zealand criteria [15, 21] using the lower IADPSG criteria [17, 19] will reduce the risk of the infant being large for gestational age (LGA).

The secondary hypotheses are that compared with the current recommended, higher New Zealand criteria [15, 21], using the lower IADPSG criteria [17, 19] will:

1. reduce the risk of serious health outcomes for the infant (composite outcome measure of perinatal death and birth trauma, defined as nerve palsy or bone fracture or shoulder dystocia);
2. reduce other infant morbidity;
3. reduce serious morbidity for the woman;
4. improve maternal psychological outcomes, quality of life and health status;
5. increase the use of induction of labour; and.
6. increase health service utilisation for the women and reduce this for the infant.

## Method/Design

### Ethics statement

Human ethics approval was granted by the Northern B Health and Disability Ethics Committee in New Zealand (13/NTB/18).

### Study design and setting

A multicentre, two-arm, parallel, randomised, controlled trial conducted at two participating hospitals in New Zealand, protocol date 2014 version 5. Women with a singleton pregnancy are eligible for the trial if they provide written, informed consent and have an OGTT for GDM at 24 to 34 weeks’ gestation. Women are not eligible if they are known to have diabetes mellitus or have previously been diagnosed with GDM.

### Trial entry, randomisation and allocation of the study group

**Fig. 1:**
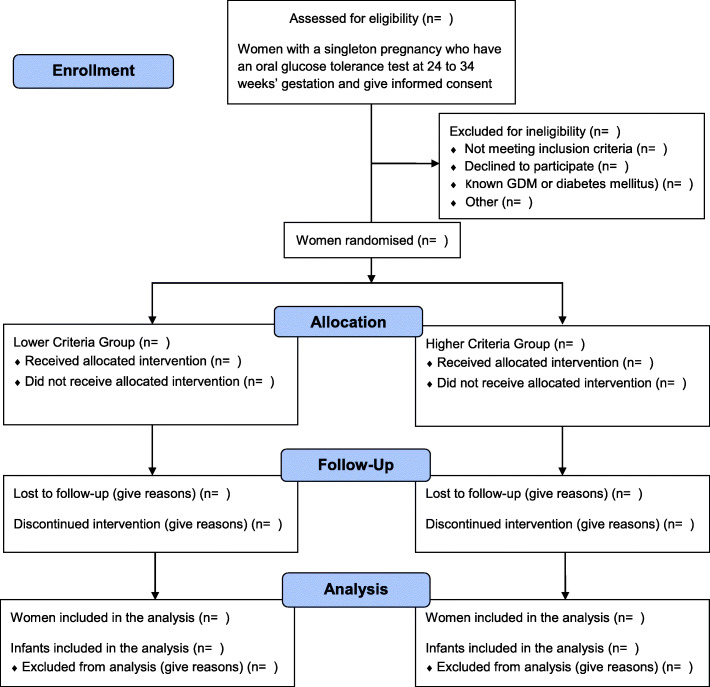
Overview of recruitment and randomisation in the GEMS Trial

Potentially eligible women will be offered participation in the GEMS Trial when considering their treatment options for testing for GDM in mid-pregnancy. They will be provided with information about the trial by their health professional or the research personnel and counselled about the study. All consenting women will have a 75 g OGTT with plasma glucose concentrations determined on fasting, and at 1 and 2 hours. After their OGTT, eligible women will be randomised by the research assistant, at a ratio of 1:1, using a central computerised system into one of two study groups: either the *Lower Criteria Group* or the *Higher Criteria Group* (Fig. 1)

.

### Generation of the sequence to which participants will be randomised

The central randomisation service will use a randomisation schedule with balanced variable blocks, prepared by an investigator not involved with recruitment or clinical care, using a randomisation table created by computer software. Stratification will be by body mass index (BMI, < 25 and ≥ 25 kg/m^2^) and by planned birthing institution.

### Treatment schedules

Women with a normal OGTT by the diagnostic criteria they are randomised to receive routine pregnancy care from their health professional. Women with GDM by the criteria they are randomised to receive standard management for GDM by their health professional and the local Diabetes Pregnancy Service, with appropriate dietary and lifestyle advice, blood glucose monitoring and further pharmacological treatment as needed [11, 21, 29]. Care of the infant after birth will be according to the hospital protocol for blood glucose monitoring.

*Women randomised to the Lower Criteria Group* therefore receive care based on their 75 g OGTT results as follows:

If the OGTT is normal by the lower criteria [19] (fasting plasma glucose concentration < 5.1 mmol/L, AND 1 hour < 10.0 mmol/L, AND 2 hour < 8.5 mmol/l) the woman receives routine pregnancy care.

If the OGTT shows GDM by the lower criteria [19] (fasting plasma glucose concentration ≥ 5.1 mmol/L, OR 1 hour ≥ 10.0 mmol/L, OR 2 hour ≥ 8.5 mmol/L) the woman receives standard care for GDM.

*Women in the Higher Criteria Group* therefore receive care based on their 75 g OGTT results as follows:

If the OGTT is normal by current criteria [15, 21] (fasting plasma glucose concentration < 5.5 mmol/L, AND 2 hour < 9.0 mmol/L) the woman receives routine pregnancy care.

If the OGTT shows GDM by the current criteria [15, 21] (fasting plasma glucose concentration ≥ 5.5 mmol/L, OR 2 hour ≥ 9.0 mmol/L) the woman receives standard care for GDM.

*In both Diagnostic Criteria Groups, w*omen, their health professionals, study personnel collecting the study outcomes and those analysing the data will be blind to the diagnostic criteria group allocated at randomisation. For the participants and their caregivers full numerical results of the OGTT will only be available after the birth.

For women with GDM in either study group with a fasting blood glucose concentration > 5.8 mmol/L or 2 hour blood glucose concentration > 11.1 mmol/L [22], the full numerical OGTT results will be released immediately to the health professional responsible for their care, as these women require urgent referral to the diabetes services.

### Data collection and management

After the birth, a research assistant at each of the participating sites will collect clinical outcome data from the case records of the participants and their infants up to the time of primary hospital discharge (Table 1). Study data will be sent to the study’s data management centre at The Liggins Institute, University of Auckland and stored in a password protected database.

**Table 1:** Schedule of enrolment, intervention, and assessments during the GEMS Trial

**Timepoint**	Enrolment	Allocation	Post-allocation			Close-out
	***Before*** ***34 weeks***	**24 to 34 weeks**	***Antenatal***	***Birth***	***Postnatal***	***Discharge after birth***
**Enrolment**						
** Eligibility screen**	X					
** Informed consent**	X					
** Allocation**		X				
**Interventions**						
** Lower Criteria Group**		X	X	X	X	X
** Higher Criteria Group**		X	X	X	X	X
**Activity/** **assessments**						
*** Inclusion/*** ***exclusion form***	X	X				
*** Consent form***	X					
*** Randomisation***		X				
*** Standard care for GDM if GDM by criteria allocated***			X	X	X	X
*** Routine pregnancy care if not GDM by criteria allocated***			X	X	X	X
*** Pregnancy, birth, and infant forms***						X
*** Serious adverse event form***			X	X	X	X
*** Primary outcome: Large for gestational age***				X	X	
*** Secondary infant outcomes***				X	X	X
*** Maternal outcomes***			X	X	X	X

### Primary study outcome

The primary study endpoint is the rate of LGA, defined as a birth weight > 90th centile using growth charts adjusted for gestational age and infant sex [30].

### Secondary study outcomes

For the infant, secondary study outcomes include other anthropometry at birth including weight, length, and head circumference and associated z-scores adjusted for gestational age and infant sex [30], LGA by customised standards [31], small for gestational age (SGA) < 10th percentile by population and customized standards; macrosomia (defined as birth weight ≥ 4 kg), gestational age at birth, preterm birth (< 37 weeks’ gestation), a composite of serious health outcomes (defined as perinatal death or birth trauma (nerve palsy or bone fracture) or shoulder dystocia) [11], Apgar score < 4 at 5 minutes, other infant morbidity including type and severity of neonatal lung disease, use of respiratory support, hypoglycaemia requiring treatment (defined as blood glucose concentration < 2.6 mmol/L), hyperbilirubinaemia requiring phototherapy, proven systemic infection in first 48 hours of life, seizures at < 24 hours age or requiring two or more drugs to control; tube feeding > 4 days; neonatal encephalopathy (Sarnat Stage 1, 2 or 3) [32], health service utilisation including neonatal intensive care admission and length of stay and length of postnatal stay.

For the woman, secondary study outcomes include a composite of serious health outcomes up to the time of primary hospital discharge after the birth [33], preeclampsia, induction of labour, mode of birth, postpartum haemorrhage (≥ 500 ml), gestational weight gain, use of pharmacological treatment for GDM, chorioamnionitis requiring antibiotics during labour, maternal infectious morbidity including puerperal sepsis requiring antibiotics, breast feeding at hospital discharge, psychological outcomes (health status [34], anxiety [35], and depression [36]), health service utilisation including health professional visits, specialist diabetes care, need for day-care admission, need for antenatal admission and length of stay, and length of postnatal stay.

### Sample size

For the primary outcome of LGA, a trial of 4,158 women will be able to show a 2.9% absolute risk reduction from 12.9% with current New Zealand criteria [15, 21] to 10.0% using the new IADPSG criteria [19], (α = 5%, two-tailed, 80% power, 10% loss to follow up), based on HAPO [22] and LGA rates from unpublished information from the ACHOIS Trial [11].

### Monitoring

The GEMS Trial Steering Group will be responsible for the conduct of the trial. An independent Monitoring Committee, with established terms of reference, will monitor the study processes and review the serious adverse events reported by the sites.

### Analysis and reporting of results

Baseline characteristics of all women randomised will be summarised descriptively by the study group allocated at randomisation, Lower Criteria Group and Higher Criteria Group, to assess comparability of the randomised groups. Treatment evaluations will use the intention-to-treat principle. Statistical tests will be two-sided and maintained at the 5% level of significance. Both unadjusted and adjusted analyses will be carried out. Secondary exploratory analyses will consider baseline covariates that show evidence of imbalance between study groups and are related to the outcome of interest. The risk estimates and 95% confidence intervals will be reported using log binomial regression for binary outcomes. Continuous outcomes will be analysed using linear regression. Statistical significance will be assessed at the 0.05 level using a two-sided comparative test.

## Discussion

Gestational diabetes is a significant and increasing health problem globally that has a major, negative effect on maternal and perinatal health, with lifelong consequences. Women with GDM are at higher risk of pre-eclampsia, more likely to have their labour induced and to give birth by caesarean section than women without GDM [4]. Their lifetime risk for developing type 2 diabetes and cardiovascular disease is high [5, 6]. Infants born to women with GDM are more likely to be LGA than infants born to women without GDM, with increased risk of an operative birth, birth injuries, and significant neonatal morbidity. Children born to women with GDM are at increased risk of being obese, developing high blood pressure and type 2 diabetes [7, 9, 37]. Treatment of women with GDM with dietary and lifestyle advice reduces adverse maternal and perinatal outcomes [11, 12] but there is still uncertainty, because of a lack of high-quality evidence, as to the optimal, diagnostic criteria to use for the diagnosis and treatment of GDM.

The GEMS Trial is assessing two diagnostic thresholds for GDM, the currently used, higher diagnostic criteria [15, 17] and the IADPSG, lower diagnostic criteria [19] for their effects on fetal growth, perinatal morbidity, maternal physical and psychological morbidity, and health service utilisation. The GEMS Trial results will establish which of these diagnostic criteria provides most benefit for maternal and infant health.

The results of the GEMS Trial will be directly relevant to the health needs of pregnant women and their infants and will provide the needed information to guide clinical practice and health policy for best care of women with GDM and their infants in New Zealand with relevance globally.

## Abbreviations

BMI: Body mass index
GDM: Gestational diabetes mellitus
OGTT: Oral glucose tolerance test

## Availability of data and materials

This manuscript consists of a trial protocol. Data sharing is not applicable as no datasets were generated or analysed in the preparation of this protocol.
